# *Rhizobium* Soaking Promoted Maize Growth by Altering Rhizosphere Microbiomes and Associated Functional Genes

**DOI:** 10.3390/microorganisms11071654

**Published:** 2023-06-25

**Authors:** Zhao Li, Yu Chi, Xianyan Su, Zhenghe Ye, Xuexiang Ren

**Affiliations:** Institute of Plant Protection and Agro-Products Safety, Anhui Academy of Agricultural Sciences, Hefei 230001, China

**Keywords:** *Bradyrhizobium japonicum*, growth, gene expression, soil microorganism, *Zea mays*

## Abstract

*Rhizobium* is a Gram-negative bacterium, which dissolves minerals, produces growth hormones, promotes root growth, and protects plants from different soil-borne pathogens. In the present study, roots, stalks, and fresh weight of maize (*Zea mays* L.) were significantly increased after soaking in *Bradyrhizobium japonicum* compared with the control. Subsequently, transcriptome sequencing results of the whole maize plant soaked in *B. japonicum* showed that multiple growth and development-related genes were up-regulated more than 100-fold compared to the control. Furthermore, the abundance of plant growth promoting bacteria, such as *Acidobacteria* Subgroup_6 and *Chloroflexi* KD4-96, were increased significantly. On the contrary, the abundance of multiple pathogens, such as *Curvularia*, *Fusarium* and *Mycocentrospora,* were significantly decreased. Moreover, inoculation with *B. japonicum* could inhibit the infection of the pathogen *Fusarium graminearum* in maize. These results suggest that soaking seeds in *B. japonicum* may affect the expression of maize growth and development-related genes as the bacteria changes the soil microorganism community structure. These findings may help to expand the application of *B. japonicum* in crop production and provide new opportunities for food security.

## 1. Introduction

Maize (*Zea may*s L.) is a cereal crop that is planted extensively worldwide [1,2]; it originated in Mexico or South America about 9000 years ago from teosinte (*Zea mays* ssp. *parviglumis*) [3,4]. Subsequently, maize was domesticated in southwestern Amazon [5]. As a staple food, animal feed, and industrial raw material, today maize has become an important part of national food security [1].

Rhizobia are Gram-negative bacteria widely distributed in soil, which infect the roots of legumes to form root nodules, fix molecular nitrogen in the air to form ammonia, provide nitrogen nutrition for plants, and have the ability to improve soil effects. Rhizobia can be divided into the α- proteobacteria class and β- proteobacteria class, of which the former mainly includes *Azorhizobium*, *Bradyrhizobium*, *Mesorhizobium*, *Rhizobium*, *Sinorhizobium*, *Methylobacterium*, *Ochrobactrum*, *Aminobacter*, *Burkholderia*, *Microvirga*, and *Phyllobacterium* [6,7,8]. Rhizobia can be extracted from a variety of crops, which is important for soil environmental improvement and agricultural production [9,10,11]. For example, rhizobia PEPV16 could produce siderophores and indole acetic acid and solubilize phosphate, as well as promote the growth of *Lactuca sativa* and *Daucus carota* [12]. Rhizobia is able to promote the growth and vitality of rice seedlings, and the benefits of early seedling development can be extended to significantly increased grain yield at maturity [13]. In addition, co-inoculation of *Rhizobia* with endophytes can increase the nodulation ability of α- and β-rhizobia, thus improving the growth of legumes [14]. The inoculation of *Rhizobium* could promote nitrogen efficiency and increase wheat yield in the Nile delta [15]. Meanwhile, Rhizobia inoculated with *Pseudomonas putida*, *P. fluorescens*, or *Bacillus cereus* can significantly increase the growth and nodulation rate of peas [16].

Soil microorganisms include microscopic organisms present in large numbers in the soil, such as bacteria, fungi, oomycetes, nematodes, protozoa, algae, archaea, and arthropods [17]. The rhizosphere soil of plants contains many microorganisms that are involved in important processes, mainly regulating plant physiology and morphology, promoting plant growth through the production of plant hormones, and acting as protective agents against plant pathogens [18,19]. For example, inoculation with plant growth promoting bacteria can ameliorate the adverse effects of salt stress on wheat yield [20]. Inoculation of *Achromobacter piechaudii* ARV8 promotes the production of ACC (1-aminocyclopropane-1-carboxylate) deaminase to alleviate drought stress in tomato and pepper [21]. In a different study, two plant growth-promoting bacteria (*B. subtilis BS87* and *B. megaterium BM89*) isolated from the sugarcane rhizosphere increased crop yield potential [22].

The aim of this study was to investigate the relationship between the growth and development of maize soaked in *Rhizobium* and the microorganism changes in maize rhizosphere soil. We found that root length, stem length, and fresh weight of maize increased significantly after rhizobia treatment. In addition, transcriptome sequencing results showed that maize growth and development-related genes were significantly up-regulated. Furthermore, rhizosphere soil microbial diversity changed significantly, and while the abundance of plant growth promoting bacteria increased significantly, the abundance of pathogenic fungi (*Fusarium* spp.) decreased significantly. Moreover, *B. japonicum* soaking could significantly reduce *Fusarium* damage to maize growth. Our results preliminarily explained the mechanism by which *Rhizobium* promoted maize growth, and provide new insights for the application of rhizobia to maize.

## 2. Materials and Methods

### 2.1. Maize, Rhizobium, and Soil

Maize seeds were provided by the Anhui Agricultural University. The soybean *Rhizobium* (*B. japonicum*) was purchased from Ruichu Biotechnology Co., Ltd. (Shanghai, China), while soil was collected from the maize fields at the Anhui Academy of Agricultural Sciences (31°53′ N, 117°14′ E).

### 2.2. Seed Treatment and Germination Test

Maize seeds were surface sterilized with 2% sodium hypochlorite for 5 min, and then washed thoroughly with autoclaved distilled water. The seeds were soaked in a *B. japonicum* solution at 25 °C for 3 h. After dressing, the seeds were air dried for 30 min at room temperature. Then, 10 seeds were sown per germination box (with soil) and were watered daily with distilled water. The amount of distilled water per treatment remained the same. Seedling length was recorded after 11 days, and root length and fresh weight were recorded after 20 days. All treatments were placed in a light incubator under the following conditions: 25 ± 1 °C, photoperiod of 16:8 h (L:D), and relative humidity of 70~80%. The *B. japonicum* soaking group was named ND-372 and the control group was named UT. Each experiment was performed with three replicates.

### 2.3. RNA Isolation, cDNA Synthesis, and Next-Generation Sequencing

We extracted RNA from the whole maize plant after 20 days. The whole samples were carried out in clean bench. Total RNA was isolated using Trizol Reagent (Invitrogen Life Technologies, Carlsbad, CA, USA), after which the concentration, quality, and integrity were determined using a NanoDrop spectrophotometer (Thermo Scientific, Waltham, MA, USA). Three micrograms of RNA were used as input material for RNA sample preparations. Sequencing libraries were generated according to the following steps. First, mRNA was purified from total RNA using poly-T oligomeric magnetic beads. Fragmentation was performed using divalent cations in Illumina proprietary fragmentation buffer at an elevated temperature. First-strand cDNA was synthesized using random oligonucleotides and Super Script II. This was followed by second-strand cDNA synthesis using DNA polymerase I and ribonuclease H. The remaining overhang was converted to blunt ends by exonuclease/polymerase activity, and the enzyme was removed. After adenylation of the 3′ ends of the DNA fragments, Illumina PE adapter oligonucleotides were ligated in preparation for hybridization. Library fragments were purified using the AMPure XP system to screen for cDNA fragments with an optimal length of 400–500 bp (Beckman Coulter, Beverly, CA, USA). DNA fragments connecting adaptor molecules at both ends were selectively enriched in 15 cycles of PCR reactions using an Illumina PCR primer cocktail. Products were purified (AMPure XP system, Beckman, Shanghai, China) and quantified using an Agilent high sensitivity DNA assay on a Bioanalyzer 2100 system (Agilent). The sequencing libraries were sequenced by Shanghai Personal Biotechnology Co., Ltd. (Shanghai, China) on a NovaSeq 6000 platform (Illumina).

### 2.4. DNA Extraction and 16S rDNA Gene Amplicon Sequencing

The rhizosphere soil samples which tightly attached to plant roots (<5 mm) from ND-372 and UT were gently collected. OMEGA Soil DNA Kit (M5635-02) (Omega Bio-Tek, Norcross, GA, USA) was used to extract the total genomic DNA samples according to the manufacturer’s instructions and stored at −20 °C until further analysis. The quantity and quality of extracted DNAs were determined using a NanoDrop NC2000 spectrophotometer (Thermo Fisher Scientific, Waltham, MA, USA) and agarose gel electrophoresis, respectively.

PCR amplification for the bacteria (338F 5′-ACTCCTACGGGAGGCAGCA-3′, 806R 5′-GGACTACHVGGGTWTCTAAT-3′) and fungi (ITS5F GGAAGTAAAAGTCGTAACAAGG, ITS2R GCTGCGTTCTTCATCGATGC) were performed. Sample-specific 7-bp barcodes were incorporated into the primers for multiplex sequencing. The PCR components included buffer (5×) 5 μL, fast pfu DNA polymerase (5 U/μL) 0.25 μL, dNTPs (2.5 mM) 2 μL, forward and reverse primer each (10 uM) 1 μL, DNA template 1 μL, and ddH_2_O 14.75 μL. Thermal cycling consisted of an initial denaturation at 98 °C for 5 min, followed by 25 cycles consisting of denaturation at 98 °C for 30 s, annealing at 53 °C for 30 s, and extension at 72 °C for 45 s, with a final extension at 72 °C for 5 min. PCR amplicons were purified using Vazyme VAHTSTM DNA Clean Beads (Vazyme, Nanjing, China). Quantification was performed via the Quant-iT PicoGreen double-stranded DNA Detection kit (Invitrogen, Carlsbad, CA, USA). Paired-end 2 × 250 bp sequencing was performed using the Illumina MiSeq platform and MiSeq Kit v3 at Shanghai Personal Biotechnology Co., Ltd. (Shanghai, China).

### 2.5. Statistical Analysis

Data were expressed as the mean ± standard deviation (SD). The data were statistically analyzed separately for each experiment using one-way ANOVA in SPSS software (v.22.0; SPSS Company, Chicago, IL, USA). Statistical significance was defined at a *p*-value of 0.05. GraphPad Prism 7 was used to plot the data in the study (GraphPad Software, Inc., San Diego, CA, USA).

HTSeq (0.9.1) statistic was used to compare the read count values of each gene as the original expression of that gene, and then FPKM was used to standardize the expression. Then, DESeq (1.30.0) was used to analyze gene differential expression; the screening conditions were as follows: expression difference multiple |log_2_FoldChange| > 1, and significant *p*-value < 0.05. At the same time, the R language Pheatmap (v.1.0.8) software package was used to perform bi-directional clustering analysis of all the different genes of samples. We calculated the distance using the Euclidian method based on the expression levels of the same gene in different samples and the expression patterns of different genes in the same sample, and cluster them using the complete linkage method to generate a heatmap.

Microbiome bioinformatics were conducted using QIIME2 2019.4 with slight modifications based on the official tutorials (https://docs.qiime2.org/2019.4/tutorials/, accessed on 16 February 2022). Sequence data analyses were mainly conducted using QIIME2 and R packages (v.3.2.0). Simply put, QIIME2 and R package (v3.2.0) were used to analyze the sequence data. The ASV table in QIME2 was used to calculate the Chao1 richness estimator, observed species, Shannon diversity index, Simpson index, Faith’s PD, Pielou’s uniformity and Good’s coverage; it was visualized in the form of block diagram. ASV sorting abundance curves were generated and the abundance and evenness of ASVs between samples were compared. Using the Jaccard metric, Bray–Curtis metric, and UniFrac distance metric, beta diversity analysis and visualized group method (UPGMA) hierarchical clustering by principal coordinate analysis (PCoA), non-metric multidimensional scale (NMDS), and unweighted arithmetic means were performed. Principal Component Analysis (PCA) was also based on general level component spectra. Multivariate analysis of variance (PERMANOVA) and similarity analysis (ANOSIM) were used to evaluate the significance of microbial community structure in the inter group differentiation. MEGAN and GraPhlAn were used to visualize their taxonomy composition and abundance. The R package “VennDiagram” was used to generate Venn diagrams to visualize samples or groups based on the occurrence of shared and unique ASVs (regardless of their relative abundance). We used MetagenomeSeq to statistically compare the taxonomic abundance of ASV levels between samples or groups and visualize it as a Manhattan map. Linear discriminant analysis effect size (LEfSe) with default parameters was used to screen out the classification groups with large differences between groups. At the same time, orthogonal partial least squares discriminant analysis (OPLS-DA) was introduced as a supervised model, and the R package “muma” was used to reveal the changes in microbiota between groups. Random forest analysis was performed using QIIME2 default settings to distinguish samples from different groups.

## 3. Results

### 3.1. B. japonicum Treatment Promoted Maize Growth

**Figure 1:**
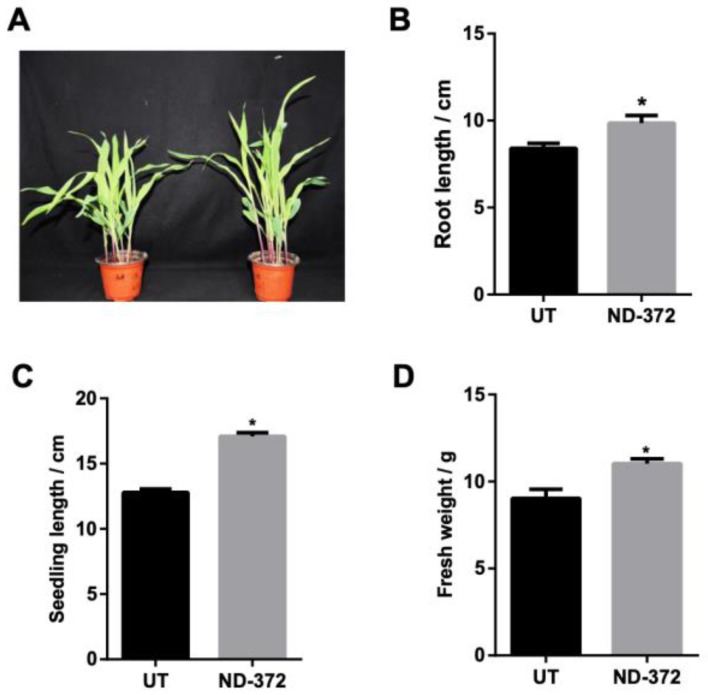
*B. japonicum* treatment promotes maize growth. (**A**) The growth situation of maize. (**B**) Root length; (**C**) Seedling length; (**D**) Fresh weight. Note: ND-372, *B. japonicum* soaking groups; UT, without *B. japonicum* soaking groups; the significant differences are marked by stars (Student’s *t*-test, *p* < 0.05).

To investigate the role of *B. japonicum* in maize growth, maize seeds were soaked in a *B. japonicum* solution (Figure 1A). Root length was significantly increased (1.45 cm on average) in treatment groups (ND-372s) compared with control groups (UTs) (Figure 1B). In addition, seedling length was also significantly increased (4.30 cm on average) in ND-372s compared with UTs (Figure 1C). Moreover, the fresh weight of ND-372 groups were significantly increased (2.00 g on average) compared with UT groups (Figure 1D). These results indicate that soaking seed in *B. japonicum* can promote maize growth.

### 3.2. The Expression of Maize Growth-Related Genes Was Induced by B. japonicum

**Figure 2:**
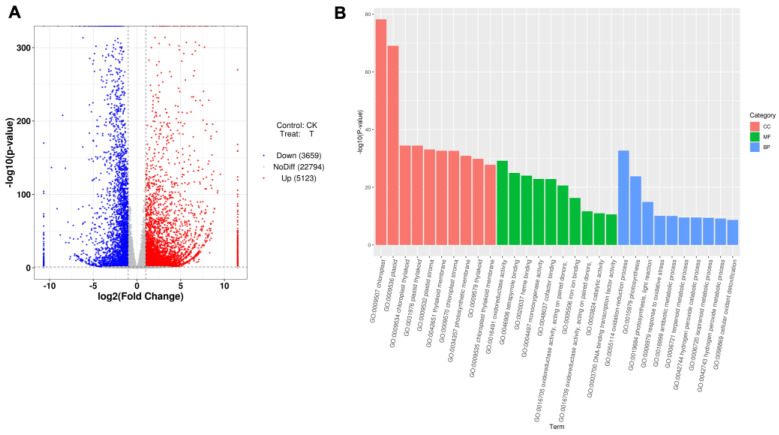
Analysis of differentially expressed genes. (**A**) Volcanic map of differentially expressed genes. The abscissa is log_2_FoldChange, and the ordinate is −log_10_ (*p*-value). The two vertical dashed lines in the figure are the 2-fold differential expression threshold. The dotted line indicates the threshold of *p*-value = 0.05. The red dots are the up-regulated genes, the blue dots are the down-regulated genes, and gray dots indicate non-significantly differentially expressed genes. (**B**) GO enrichment analysis of differentially expressed genes. The abscissa is the term of Go level2, and the ordinate is the enriched −log_10_ (*p*-value) of each term.

In order to explore the role of *B. japonicum* in promoting maize growth, transcriptome sequencing was performed. First, the sample correlation test and PCA analysis showed that the correlation between the treatments was appropriate in both the UT (CK, untreated) and ND-372 (T, treatment) groups, respectively (Figure S1A,B). Second, gene differential expression analysis indicated that 8782 genes were differentially expressed: 5123 were up-regulated and 3659 were down-regulated (Table 1, Figure 2A). In addition, GO enrichment analysis showed that differentially expressed genes were distributed in molecular functions, biological processes, and cell components (Figure 2B). Furthermore, a total of 16 genes related to growth and development were significantly up-regulated more than 20-fold (Table 2). It was reasonable to speculate that *B. japonicum* treatment was related to maize growth and development.

### 3.3. B. japonicum Treatment Increased the Abundance of Maize Rhizosphere Bacteria

To reveal the role of soil bacteria on maize growth, an Ilumina HiSeq PE250 platform was used to investigate bacteria changes in soil. In total, 527,312 valid sequences and 25,071 operational taxonomic units (OTUs) were detected in eight groups (Table 3). All UT and ND_372 samples contained 4966 OTUs, and conventional OTUs accounted for nearly 19.81%, while the remaining 37.23% and 42.97% were found in UT and ND_372, respectively (Figure S2A).

**Figure 3:**
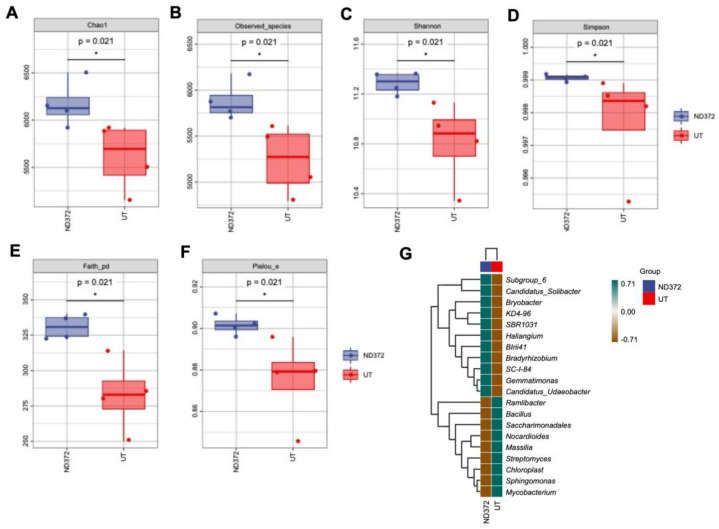
Alpha diversity index, species differences and marker species of rhizosphere soil bacteria (**A**) Chao1; (**B**) Observed_species; (**C**) Shannon; (**D**) Simpson; (**E**) Faith_pd; (**F**) Pielou_e. Each panel corresponds to an alpha diversity index, identified in the gray area at the top. In each panel, the abscissa is the group label, and the ordinate is the value of the corresponding alpha diversity index. (**G**) Species differences and marker species of rhizosphere soil bacteria. The samples were clustered according to the Euclidean distance of species composition data (the default clustering algorithm) via UPGMA, and arranged according to the clustering results by default. The significant differences are marked by stars (Student’s *t*-test, *p* < 0.05).

In order to explore the relationship between soil bacteria and maize growth, we conducted bacteria alpha diversity analysis. Compared with the UT, the observed species values and Chao1 diversity index were higher in the ND_372 groups (Figure 3A,B), indicating that ND_372 had a greater richness. The Shannon and Simpson’s diversity indexes were increased significantly, indicating that bacterial community diversity and evenness in the ND_372 groups were higher than that in UT groups (Figure 3C,D). In addition, Pielou’s evenness and Good’s coverage index were also increased significantly, which indicated a higher level of diversity evolution and evenness (Figure 3E,F). To evaluate the contribution of bacteria, genus composition analysis was performed. The results showed that the abundance of many plant growth promotion-related bacteria, such as subgroups_6 and KD4-96, increased significantly (Figure 3G). The results suggested that soaking seed in *B. japonicum* may promote the growth of maize by altering the abundance of bacteria.

### 3.4. B. japonicum Treatment Affected the Variation of Maize Rhizosphere Fungi

To explore the effects of soil fungi on maize growth, 705,404 valid sequences and 1324 operational taxonomic units (OTUs) were detected in eight groups (Table 4). All UT and ND_372 samples contained 310 OTUs, and conventional OTUs accounted for nearly 23.42%, while the remaining 20.24% and 54.98% were found in UT and ND_372, respectively (Figure S2B).

**Figure 4:**
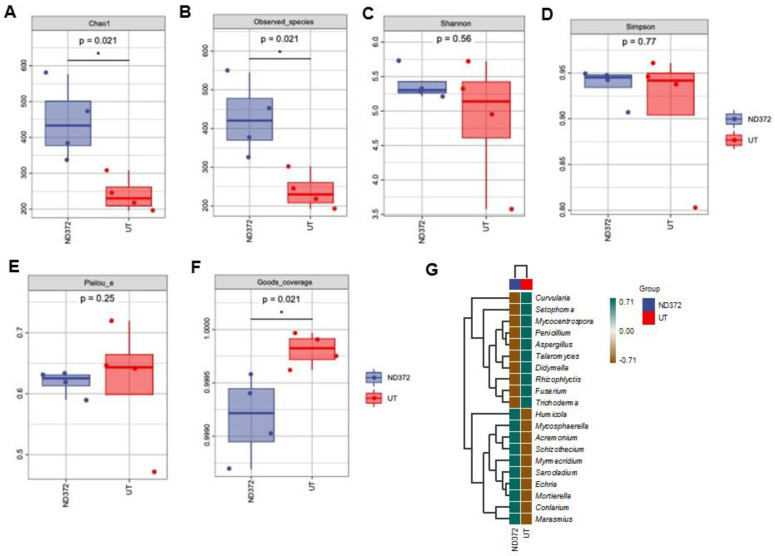
Alpha diversity index, species differences and marker species of rhizosphere soil fungi. (**A**) Chao1; (**B**) Observed_species; (**C**) Shannon; (**D**) Simpson; (**E**) Faith_pd; (**F**) Pielou_e. Each panel corresponds to an alpha diversity index, identified in the gray area at the top. In each panel, the abscissa is the group label, and the ordinate is the value of the corresponding alpha diversity index. (**G**) Species differences and marker genera of rhizosphere soil fungi. The samples were clustered according to the Euclidean distance of genera composition data (the default clustering algorithm) via UPGMA, and arranged according to the clustering results by default. The significant differences are marked by stars (Student’s *t*-test, *p* < 0.05).

**Figure 5:**
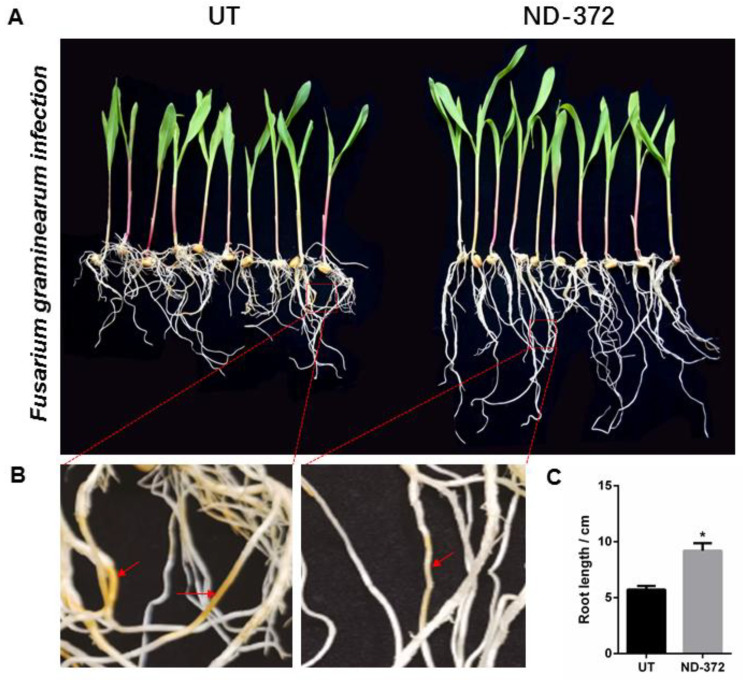
*B. japonicum* treatment inhibit the infection of *Fusarium* on maize roots. (**A**,**B**) The left is *Fusarium* infestation of maize roots; and the right is *Fusarium* infestation of maize roots with *B. japonicum* infiltration. (**C**) Root length. The significant differences are marked by stars (Student’s *t*-test, *p* < 0.05).

In order to explore the relationship between soil microorganisms and maize growth, we conducted a fungal alpha diversity analysis. Compared with the UT, the observed species values and Chao1 diversity index were higher in ND_372 (Figure 4A,B), indicating that ND_372 had greater richness. There were no significant differences between UT and ND_372 in Shannon, Simpson’s diversity, or Pielou’s evenness indexes, indicating that the microbial community diversity and evenness were similar (Figure 4C–E). However, the Good’s coverage index of the UT group was significantly higher than that of the ND_372 treatment group, indicating that the UT group had better coverage (Figure 4F). To evaluate the contribution of fungi, species composition analysis was performed. The results showed that the abundance of many kinds of microorganisms changed, among which the pathogenic fungi *Curvularia*, *Mycocentrospora* and *Fusarium* decreased significantly (Figure 4G). Moreover, inoculation of rhizobia could significantly reduce the infection of *Fusarium graminearum* on maize root (Figure 5A,B). Meanwhile, the root length was significantly increased (3.48 cm on average) in treatment groups (ND-372) compared with the control groups (UT) (Figure 5C). The results suggest that *B. japonicum* treatment changed the soil fungal microbial environment, which may inhibit the pathogenic fungi (e.g., *Fusarium* species).

## 4. Discussion

Rhizobia are Gram-negative bacilli that exist widely in soil and play an important role in agricultural production [23]. Previous studies have shown that rhizobia are associated with promoting plant growth and improving plant resistance to stress. For example, *Rhizobium leguminosarum,* isolated from non-leguminous plants, is reported to promote rice growth [11]. Furthermore, dual-inoculation of *Rhizobium* and mycorrhizal fungi reduced drought stress and increased the relative water content, fat content, and yield of soybean plants [24]. But the effects of *B. japonicum* on maize growth and rhizosphere soil microorganisms have rarely been reported. In the present study, *B. japonicum* infiltration was shown to alter the structure of rhizosphere soil microorganisms and to have played a role in regulating the growth and development of maize. In this study, we found that the typical growth indexes, including the root length, seedling length, and fresh weight of maize were significantly increased following *B. japonicum* treatment. Similarly, co-inoculation with *MesoRhizobium ciceris*, *Pseudomonas* sp., and *Bacillus* sp. significantly increased seed germination and shoot and root length compared with an uninoculated control in *Cicer arietinum* [25]. It is therefore reasonable to speculate that *B. japonicum* is involved in promoting maize growth.

Genes contain complete sequences of nucleotides required to produce a polypeptide chain or functional RNA, which notably affect living processes in organisms. In crops, a large number of genes are known to be involved in regulating crop growth and morphological characters. For instance, functions of the *TaYUCCA* gene family are diverse, and may play an important role in the growth and development of wheat [26]. *Gmpskγ1* is a novel *PSK* encoding gene in soybean, which promotes cell expansion in transgenic plants, thereby increasing seed size and yield [27]. *OsGASR9* promotes germination, width, and thickness of rice, while *Ghgasa10-1* promotes seedling germination and root elongation of transgenic *Arabidopsis thaliana*, indicating that *GhGASA10-1* promotes cell elongation [28]. Recent studies have shown that knockout of *KRN2* in maize or *OsKRN2* in rice increases grain yield by ~10% and ~8%, respectively [29]. The *Tacol-b5* gene regulates spikelet number, tiller number, and yield per plant in wheat, and field yield tests show that *Tacol-b5* significantly promotes wheat yield [30]. Here, we found that several growth and development-related genes of maize, including *Zm00001eb397820*, *Zm00001eb125420*, *Zm00001eb244140*, *Zm00001eb204390*, and *Zm00001eb147260* were significantly up-regulated by >100-fold under *B. japonicum* treatment, suggesting that *B. japonicum* infiltration promotes the growth and development of maize. Similar to our results, RNA-seq results from Zhang et al. show that the expression of *Zm00001d005892*, *Zm00001d027925*, *Zm00001d047017*, and *Zm00001d039245* are related to root architecture or development in arabidopsis, rice, and maize [31].

The rhizosphere is a narrow zone that is around and affected by plant roots; a large number of microorganisms exist in this region and it is considered as one of the most complex ecosystems [32]. The rhizosphere soil microbial community is considered an indicator of soil quality and yield, which greatly affects the formation of soil fertility and the transformation of plant nutrition. A stable soil microbial community is very important to agricultural ecosystems and is conducive to maintaining the stability of soil systems [33]. Plant rhizosphere bacteria (PGPR) are bacteria that promote plant growth through a variety of mechanisms, including phosphate dissolution, siderophore production, biological nitrogen fixation, and rhizosphere engineering [34]. A number of PGPR have been applied, for example, *Pseudomonas*, *Bacillus*, *Enterobacter*, *Klebsiell*a, *Azobacter*, *Variovorax Azosprillum*, and *Serratia* [35]. In our study, the abundance of multiple plant growth-promoting bacteria, such as *Acidobacteria* subgroup_6 and *Chloroflexi*_KD4-96, increased significantly. Consistent with our results, inoculation with *SinoRhizobium* sp. A15, *Bacillus* sp. A28, and *Sphingomonas* sp. A55 increased the abundance and species richness of maize rhizosphere bacteria; moreover, the relative abundances of *Acidobacteria* subgroup_6, *Chloroflexi*_KD4-96, *Verrucomicrobiae*, and *Mucilaginibacter* at the genus level were positively correlated with maize biomass and yield [36]. In addition, the abundance of many types of microorganisms changed, among which the pathogenic fungi *Curvularia* and *Fusarium* decreased significantly. Previous studies have demonstrated that *Fusarium* head blight (FHB), caused by the fungal pathogen *F. graminearum*, is an important economic disease of wheat and also causes panicle rot and stem rot verticillium wilt that seriously affect maize yield and quality [37,38]. Our study showed that soaking maize seeds in *B. japonicum* could increase the abundance of growth-promoting bacteria and reduce the abundance of pathogenic fungi in soil, which may greatly improve maize production.

## 5. Conclusions

In conclusion, this work reveals that *B. japonicum* treatment is able to promote maize growth by inducing significant expressions of growth-associated genes in maize. We also found that *B. japonicum* treatment remodels the rhizosphere microbiomes of maize. Thus, our work provides a novel and efficient method to expand the application of *B. japonicum* in crop production and provide new opportunities for food security in an environmentally friendly manner.

## Figures and Tables

**Table 1:** Number of differentially expressed genes.

Control	Treat	Up-Regulated	Down-Regulated	Total
UT	T (ND-372)	5123	3659	8782

**Table 2:** Significantly up-regulated genes related to growth and development of seed soaked in *B. japonicum*.

Gene ID	Foldchange (ND-372/UT)	Potential Function
Zm00001eb397820	433.63	multicellular organism development
Zm00001eb125420	168.58	multicellular organism development
Zm00001eb244140	167.19	positive regulation of organ growth
Zm00001eb204390	136.68	vegetative to reproductive phase transition of meristem
Zm00001eb147260	105.73	structure development
Zm00001eb123060	88.90	multicellular organism development
Zm00001eb054040	67.45	oxidation-reduction process of development
Zm00001eb074640	56.91	response to auxin
Zm00001eb236120	56.38	meristem initiation
Zm00001eb110420	56.36	plant organ development
Zm00001eb248500	45.89	syncytium formation
Zm00001eb243730	33.32	system development
Zm00001eb330990	29.49	lateral root formation
Zm00001eb335320	28.74	cytokinin-activated signaling pathway
Zm00001eb308610	21.35	embryo development ending in seed dormancy
Zm00001eb012940	21.09	multicellular organism development

**Table 3:** General statistics of bacteria via the 16S rDNA sequencing.

SampleID	Input	Filtered	Denoised	Merged	Nonchimeric	Nonsingleton
ND372_1	134,425	125,195	116,500	80,010	67,478	62,262
ND372_2	147,860	1365,91	126,988	84,603	69,399	63,651
ND372_3	146,836	129,701	120,458	79,713	65,519	60,463
ND372_4	138,138	128,174	120,050	82,681	69,203	64,372
UT_1	138,595	128,379	121,750	90,777	75,797	72,260
UT_2	135,707	124,918	118,907	91,637	74,928	71,524
UT_3	141,972	130,331	122,674	87,220	72,159	68,014
UT_4	146,266	134,626	126,237	87,319	69,539	64,766

**Table 4:** General statistics of fungi via the 16S rDNA sequencing.

SampleID	Input	Filtered	Denoised	Merged	Nonchimeric	Nonsingleton
ND_372_1	192,013	153,449	152,606	150,526	148,915	148,915
ND_372_2	152,905	129,712	128,835	127,487	124,810	124,809
ND_372_3	108,237	81,117	80,415	79,215	77,885	77,885
ND_372_4	120,495	102,122	101,522	100,854	97,385	97,383
UT_1	88,801	77,634	77,138	75,670	72,733	72733
UT_2	89,368	63,851	63,321	61,711	56,830	56,830
UT_3	96,514	82,242	81,509	80,691	78,083	78,083
UT_4	64,372	53,757	53,034	52,104	48,766	48,766

## Data Availability

Data will be made available on request.

## Supplementary Materials

The following supporting information can be downloaded at: https://www.mdpi.com/article/10.3390/microorganisms11071654/s1, Figure S1: Correlation test; Figure S2: Summary of different species and markers in rhizosphere soil bacteria.
