# Supplementary material for: Rhizobium Soaking Promoted Maize Growth by Altering Rhizosphere Microbiomes and Associated Functional Genes
Source: Microorganisms. 2023 Jun 25;11(7):1654. doi: 10.3390/microorganisms11071654 (PMC10383385; doi:10.3390/microorganisms11071654)
Supplement: Supplementary file 1 [file microorganisms-11-01654-s001.zip › microorganisms-2439488-supplementary.pdf]

Supplementary Material

Supplementary Figures

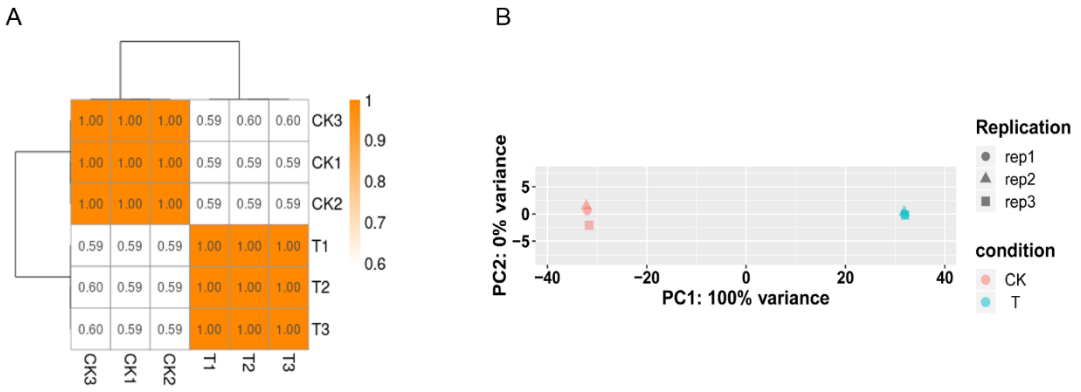

Figure S1 Correlation test. (A) Sample correlation test. The left and upper sides are sample clustering, and the right and lower sides of the figure are sample names. Squares with different colors represent the correlation between the two samples. **(B) Principal Components Analysis.** The abscissa is the first principal component, and the ordinate is the second principal component. Different shapes represent different samples, and different colors represent different groups.

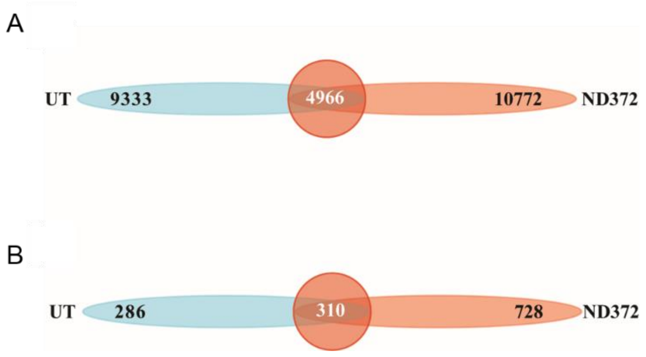

Figure S2 Summary of different species and markers in rhizosphere soil bacteria. (A) Rhizosphere soil bacteria; (B) Rhizosphere soil fungi. Each color block represents a group, the overlapping area between the color blocks indicates the OTUs shared

between the corresponding groups, and the number of each block indicates the number of OTUs contained in the block.
